# Screening and verification of long noncoding RNA promoter methylation sites in hepatocellular carcinoma

**DOI:** 10.1186/s12935-020-01407-4

**Published:** 2020-07-15

**Authors:** Zhuo Lin, Xiaofeng Ni, Shengjie Dai, Hao Chen, Jianhui Chen, Boda Wu, Jianyang Ao, Keqing Shi, Hongwei Sun

**Affiliations:** 1 Department of Liver Diseases, The First Affiliated Hospital of Wenzhou Medical University, Wenzhou, Zhejiang Province People’s Republic of China; 2 Key Laboratory of Diagnosis and Treatment of Severe Hepato-Pancreatic Diseases of Zhejiang Province, The First Affiliated Hospital of Wenzhou Medical University, Wenzhou, Zhejiang Province People’s Republic of China; 3 Department of Surgery, The First Affiliated Hospital of Wenzhou Medical University, Wenzhou, Zhejiang Province People’s Republic of China; 4 Chinese Academy of Sciences Shanghai Branch, Shanghai, People’s Republic of China; 5 Laboratory of Precision Medicine Center, The First Affiliated Hospital of Wenzhou Medical University, Wenzhou, Zhejiang Province People’s Republic of China

**Keywords:** LncRNA, Hepatocellular carcinoma, Bioinformatics, Promoter methylation, Overall survival, Vascular invasion

## Abstract

**Background:**

Long noncoding ribonucleic acid (lncRNA) promoter methylation is closely related to the occurrence and development of hepatocellular carcinoma (HCC). Thus, we aim to screen and verify the lncRNA promoter methylation sites associated with overall survival (OS), vascular invasion, pathological grade, and clinical stage in HCC.

**Methods:**

Methylation-related data including clinical characteristic, transcriptome, methylation, and messenger RNA (mRNA) expression were taken from the Cancer Genome Atlas (TCGA) database. The OS, vascular invasion, pathological grade, and clinical stage-related lncRNA promoter methylation models were developed by the least absolute shrinkage and selection operator (LASSO) algorithm based on the lncRNA promoter methylation sites screened via R software. The Kaplan–Meier analysis, the area under the receiver operating characteristic (ROC) curve (AUC), the calibration curve (C-index) were performed to evaluate the performance of these models. Finally, the methylation-specific polymerase chain reaction (MS-PCR) was performed to verify the accuracy of these models based on 146 HCC tissues from our hospital.

**Results:**

A total of 10 methylation sites were included in the OS-related lncRNA promoter methylation model that could effectively divide HCC patients into high-risk and low-risk groups (P < 0.0001) via survival analysis. COX univariable and multivariable regression analysis found that the OS-related model (P < 0.001, 95% CI 1.378–2.942) and T stage (P < 0.001, 95% CI 1.490–3.418) were independent risk factors affecting OS in HCC patients. The vascular invasion-related model contained 8 methylation sites with its AUC value of 0.657; the pathological grade-related model contained 22 methylation sites with its AUC value of 0.797; the clinical stage-related model contained 13 methylation sites with its AUC of 0.724. Target genes corresponded to vascular invasion-related lncRNA promoter methylation sites were involved in many kinds of biological processes in HCC such as PI3K-Akt signaling pathway. The accuracy of the vascular invasion-related model was consistent with our bioinformatics conclusion after being verified via MS-PCR.

**Conclusion:**

The lncRNA promoter methylation sites are closely correlated with the process of HCC and can be utilized to improve the therapy and prognosis of HCC.

## Background

HCC is the most common type of primary liver cancer (PLC) [1]. Because of the poor diagnostic approaches and the high recurrence and metastasis rates, the 5-year survival rate is still at a low level [2, 3]. Thus, it is extremely important to identify biomarkers that are specific and sensitive for HCC [4].

With the breakthrough of high-throughput sequencing technology, the biological functions of noncoding RNAs are being discovered gradually [5], and their abnormal functioning can lead to the occurrence of neoplasms [6]. Among ncRNAs, lncRNAs play a critical role in the occurrence and development of HCC, and they are involved in proliferation, differentiation, metastasis, invasion, apoptosis, and metabolism [7] and have become noval field of cancer biology in recent years [8]. Some of the lncRNAs that have been reported to be associated with HCC so far include HULC [9], HOTAIR [10], MALAT1 [11], H19 [12], and so on. Additionally, lncRNAs found in serum have been demonstrated to be potential blood-based noninvasive markers for clinical and therapeutic targets of HCC [13]. Xiao et al. [14] identified that lncRNAs associated with prognosis could be used as biomarkers for predicting the OS of HCC patients.

Furthermore, the common patterns of lncRNA regulation of cellular physiological are as follows: lncRNA regulates the target gene by affecting gene epigenetics such as the promoter methylation [15]; and lncRNA competes with miRNA for its target RNA, making the miRNA unable to affect the function of the target mRNA [16, 17]. Recent studies have found that the expression levels of CDKN2A, HHIP, PTGR1, TMEM106A, MT1M, MT1E, and CPS1 in HCC tissues are significantly reduced, and this is caused by its promoter region methylation. These genes are involved in various processes of HCC [18]. Another study found that the expression of ZEB1-AS1 is significantly increased because its promoter is hypomethylated, which leads to patients with these manifestations having a poor prognosis [19].

Based on the significance of promoter methylation for HCC-related lncRNA expression regulation and molecular mechanisms, we utilized bioinformatics methods to screen lncRNA promoter methylation sites that were associated with the occurrence and development of HCC. Then, a total of 146 samples of HCC tissues resected from HCC patients at our hospital were utilized to verify the methylation degree of the prescreened lncRNA promoter methylation sites and verify the accuracy of the models constructed above, which was to determine the HCC-specific lncRNA promoter methylation sites.

## Materials and methods

### Research object

The clinical characteristic, transcriptome, methylation, and mRNA data were taken from the TCGA database. The exclusion criteria were as follows: histopathological diagnosis in not HCC; and sample data are incomplete. A total of 367 cases were eligible for this study. Additionally, we collected 146 HCC tissues with their clinical characteristics from the Central Laboratory Human Specimen Library from HCC patients who underwent surgical therapy between June 2017 and April 2018 at our hospital. The collection of the 146 HCC samples was approved by the Central Laboratory Human Specimen Library. This study was approved by the Review of Ethics Committee in Clinical Research (ECCR) of the First Affiliated Hospital of Wenzhou Medical University according to the Regulations and Rules of “Ethical Reviews for Biomedical Research Involving Human Subjects” (2016) of the National Health Commission of PRC, “Declaration of Helsinki” of WMA, and “International Ethical Guidelines for Human Biomedical Research” of CIOMS.

### Screening of the lncRNA promoter methylation sites

The methylation sites related to lncRNA were screened from lncRNA promoter region located within 2 kb upstream of transcription start site (TSS) according to the annotation of methylation sites by using a HumanMethylation450 chip. After analyzing the beta values normalized by the methylation chip data, the methylation sites with a significant difference between HCC and adjacent tissues and an absolute value of the beta difference greater than 0.1 were selected. The final methylation sites with a significant negative correlation between the beta value and lncRNA expression were screened for further analyzing.

### Cluster analysis and heatmap production

The beta values of each lncRNA promoter methylation site screened in the previous experiments were normalized by Z-scores. Then, R software was utilized to construct a heatmap with the “pheatmap” package.

### LASSO regression to develop the lncRNA promoter methylation models

We utilized the “glmnet algorithm” package in R software to establish LASSO-Logistic and LASSO-COX regression classification models [20]. Construction of lncRNA promoter methylation models by LASSO-COX algorithm were used for evaluating the OS of HCC patients; construction of lncRNA promoter methylation models for evaluating vascular invasion, pathological grade, and clinical stage used the LASSO-Logistic algorithm.

### Evaluation and analysis of the lncRNA promoter methylation models

The ROC curves constructed by MedCalc (version 14.0) were used to evaluate the accuracy of the different lncRNA promoter methylation models associated with vascular invasion, pathological grade, and clinical stage. A time-dependent ROC (tdROC) analysis performed by the SurvivalROC program in R software was used to evaluate the accuracy of the OS-related lncRNA promoter methylation model. The recognition ability of the ROC curves and the tdROC curves were evaluated by AUC values. COX regression analysis was performed to determine whether the OS-related lncRNA promoter methylation model was a separate risk factor for predicting the OS of HCC patients. The Kaplan–Meier survival analysis of the OS-related lncRNA promoter methylation model was performed to divide HCC patients with the following categories of OS, tumor-free status, pathological grade, clinical stage, age (the boundary of 65 years), and sex into high-risk and low-risk groups.

### The functional analysis of lncRNA corresponding to vascular invasion-related lncRNA promoter methylation sites

Co-expression analysis between lncRNA and mRNA was utilized to screen for mRNAs positively associated with lncRNA for functional analysis. The function of the lncRNA was analyzed by utilizing “clusterProfiler” package in R software for KEGG pathway and GO analysis, and revealed differentially expressed genes involved in tumor-associated signaling pathways.

### Genomic DNA extraction and bisulfite conversion assay in HCC tissues

A TIANamp Genomic DNA Kit (TIANGEN, Beijing, China) was utilized to extract total genomic DNA from the HCC tissues. Then, an EZ DNA Methylation-Gold Kit (ZYMO Research, USA) was utilized to perform bisulfite conversion with a certain mass of DNA (500 ng) for subsequent methylation-specific PCR (MSP).

### Methylation-specific PCR assay

After extraction of 146 DNA samples and bisulfite conversion, real-time quantitative PCR (qRT-PCR) experiments were performed by utilizing a FAST qPCR Master Mix (2×) Kit (KAPA Biosystems, Wilmington, Massachusetts, USA) to detect the methylation degree of HCC tissues. First, a total of 146 DNA samples were divided into two groups, the vascular invasion group (n_1_ = 66) and the nonvascular invasion group (n_2_ = 80), based on their pathological results. Then, DNA samples with its reaction system solution used one set of 96-well PCR plates (Applied Biosystems, Thermo Fisher Scientific, USA), which were read on a 7500 FAST Real-Time Fluorescent PCR System (Applied Biosystems, Thermo Fisher Scientific, USA). The primers used here were designed and synthesized by Invitrogen (Thermo Fisher Scientific, USA), and their sequences are shown in Table 1. The results were analyzed by the 2^−ΔΔCt^ method.

**Table 1:** List of the methylation-specific PCR primer sequence

CG sites	Sequence
cg11201447	
Forward	AATTTGATATAGTTTTGTGGTTATAGC
Reverse	AATTCTATATTTATCCTTCTACAACTTCC
cg16186435	
Forward	TTTTTAGTTTTTGGGTGGGGAC
Reverse	CCACTACTACAATCACTCCATACT
cg16201808	
Forward	GGCGTTAGAGTGGATATTGC
Reverse	ATATACCTTTTACCTTCTACCATAATC
cg20535723	
Forward	AGTTAGTGGGGAGTGAGGTC
Reverse	CCAATCTTACAACTTTCTAAAATAACAAAT
cg03209812	
Forward	GTTTGTGGTAGAAAAATCGAGTTTAAGT
Reverse	TATAATAACGCTTCCCCTCTCCTAA
cg14743534	
Forward	TAGCGGTGGGTGGGGTCG
Reverse	AAACTTCATCACCAAACTCGTAAACAT
ACTB	
Forward	TGGTGATGGAGGAGGTTTAGTAAGT
Reverse	AACCAATAAAACCTACTCCTCCCTTAA

### Statistical analysis

The data analysis was completed by utilizing R version 3.5.3 software (University of Science and Technology of China). For the methylation-specific PCR assay, we adjusted the fluorescence threshold to 0.56 to normalize all of the samples. Additionally, a *P* value less than 0.05 was considered to have statistical significance.

## Results

### Baselines clinical characteristics of the HCC samples

Among 367 cases of HCC patients in the TCGA database, 249 patients were men (67.8%) and 118 patients were women (32.2%); the average age of the patients was 59.6 ± 13.3 years old; 131 patients (35.7%) died, and 236 patients survived; and 197 patients (53.7%) currently had a tumor-free status. The baseline of the 146 HCC patients’ clinical characteristics were shown in Table 2. Among them, 50 patients were older than 65 years (34.2%), and the majority of patients were men (87.0%). A total of 66 HCC tissues had vascular invasion according to pathological grade, and the majority of the HCC tissues were at pathological grade II.

**Table 2:** Baseline of clinical characteristic of HCC patients

Characteristic	Patients (%)
All patients (n = 146)	
Age	
< 65	96 (65.8)
≥ 65	50 (34.2)
Gender	
Male	127 (87.0)
Female	19 (13.0)
Pathological grade	
I	19 (13.0)
II	77 (52.7)
III	26 (17.8)
IV	24 (16.5)
Vascular infiltration	
Positive	66 (45.2)
Negative	80 (54.8)

All values are presented as quantity and percentage of cases

### Development of the OS-related lncRNA promoter methylation model

**Fig. 1:**
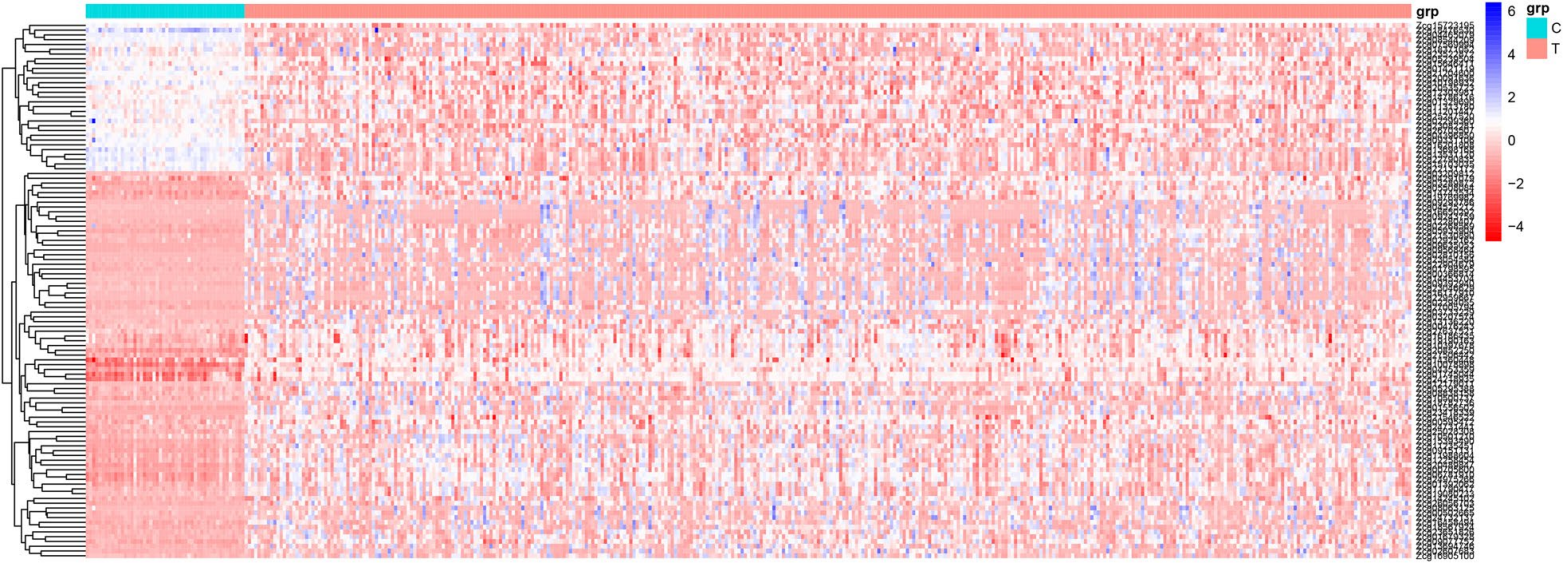
Differential expression heatmap of lncRNA promoter methylation sites between HCC cancer tissues and adjacent tissues

**Fig. 2:**
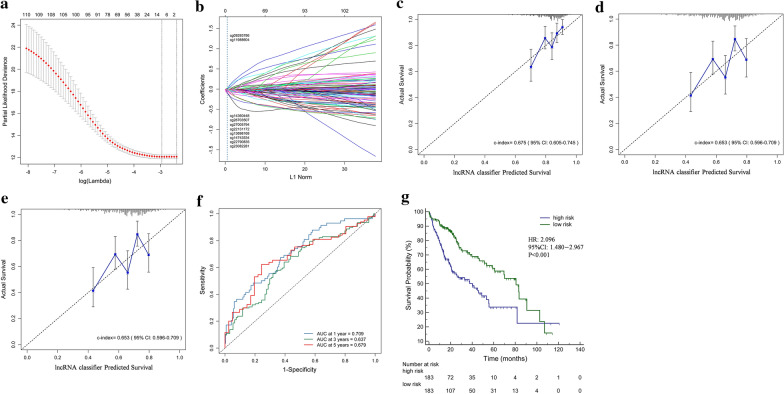
Development and evaluation of the OS-related lncRNA promoter methylation model. **a** OS-related lncRNA promoter methylation sites were screened by LASSO-COX method. **b** The coefficients of each methylation sites were determined by LASSO-COX method. **c** The calibration curve of predicted 1-year OS of HCC patients by OS-related lncRNA promoter methylation model. **d** The calibration curve of predicted 3-year OS of HCC patients by OS-related lncRNA promoter methylation model. **e** The calibration curve of predicted 5-year OS of HCC patients by OS-related lncRNA promoter methylation model. **f** ROC curve of predicted 1-, 3-, and 5-year OS of HCC patients by OS-related lncRNA promoter methylation model. **g** The Kaplan–Meier analysis of OS-related lncRNA promoter methylation model for HCC patients from the TCGA database

There were 112 methylation sites identified with a significant negative correlation between the beta values and the expression of lncRNA. To visualize the results above, a heatmap was constructed and was shown in Fig. 1. The LASSO-COX algorithm was performed to select the variables, determine the coefficients, and finally derive the OS-related lncRNA promoter methylation model (Fig. 2a, b). In Fig. 2a, the best lambda value is at the lowest point of the red curve (i.e., at the dotted line) with 10 lncRNA promoter methylation sites.

The OS-related lncRNA promoter methylation model was constructed as follows:

$$ \begin{aligned} & 0.0 3 2\, \times \,{\text{E}}\_{\text{cg}}0 9 2 9 3 7 8 6\, + \,0.0 1 7\, \times \,{\text{E}}\_{\text{cg119886}}0 4\, - \,0.0 5 5\\ & \quad \times \,{\text{E}}\_{\text{cg13698168}}\, - \,0.00 1\, \times \,{\text{E}}\_{\text{cg1436}}0 4 4 8\, - \,0.0 7 4\\ & \quad \times \,{\text{E}}\_{\text{cg14743534}}\, - \,0.0 5 4\, \times \,{\text{E}}\_{\text{cg22131172}}\, - \,0.0 8 5\\ & \quad \times \,{\text{E}}\_{\text{cg2279}}0 8 3 5\, - \,0. 10 8\, \times \,{\text{E}}\_{\text{cg23}}0 8 2 2 8 1\, - \,0.00 5\\ & \quad \times \,{\text{E}}\_{\text{cg267}}0 3 50 7\, - \,0.0 2 7\, \times \,{\text{E}}\_{\text{cg27}}00 5 7 9 4. \\ & {\text{E}}\_{\text{cg}}\; = \,\beta {\text{ value }}\left( {\text{the degree of lncRNA promoter methylation}} \right). \\ \end{aligned} $$

### Evaluation of the OS-related lncRNA promoter methylation model

In Fig. 2c–e, the calibration curves suggested that the OS-related model possesses a certain accuracy better than random guessing, with their C-indexes for predicted 1-, 3-, and 5-year OS were 0.675 (95% CI 0.605–0.745), 0.653 (95% CI 0.596–0.709), and 0.651 (95% CI 0.597–0.706), respectively. The results of the td-AUC values of the td-ROC curve for the predicted 1-, 3-, and 5-year OS were 0.709 (95% CI 0.635–0.784, sensitivity: 67.40%, specificity: 63.08%, accuracy: 63.66%), 0.637 (95% CI 0.557–0.716, sensitivity: 68.35%, specificity: 56.38%, accuracy: 59.84%), and 0.679 (95% CI 0.585–0.773, sensitivity: 62.35%, specificity: 75.61%, accuracy: 72.22%), respectively, which could derive the same conclusion for the C-index (Fig. 2f). In Fig. 2g, this OS-related model could effectively divide HCC patients into a high-risk group and a low-risk group, and the survival rate of the high-risk group was significantly lower than the low-risk group (HR: 2.096, 95% CI 1.480–2.967, P < 0.001).

**Fig. 3:**
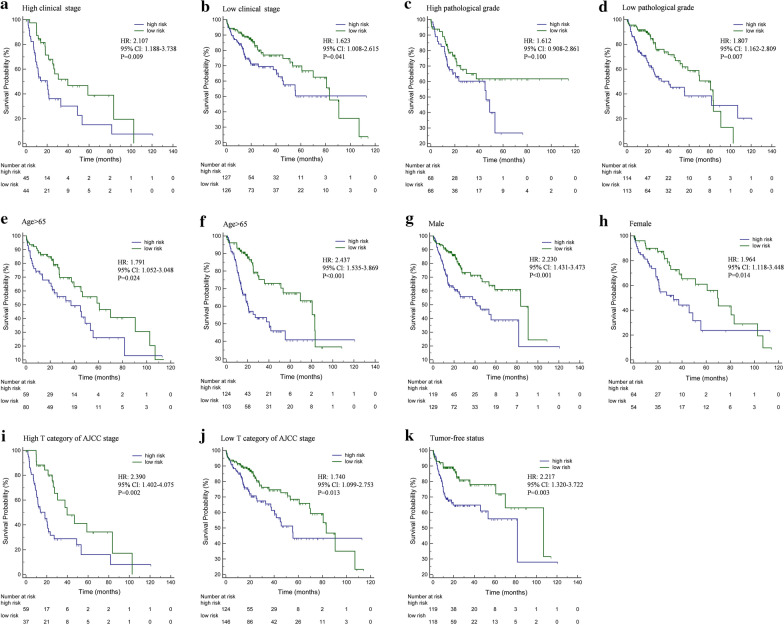
The Kaplan–Meier analysis of grouped HCC patients by OS-related lncRNA promoter methylation model. **a** The survival analysis of HCC patients with high tumor clinical stage by OS-related lncRNA promoter methylation model. **b** The survival analysis of HCC patients with low tumor clinical stage by OS-related lncRNA promoter methylation model. **c** The survival analysis of HCC patients with high pathological grade by OS-related lncRNA promoter methylation model. **d** The survival analysis of HCC patients with low pathological grade by OS-related lncRNA promoter methylation model. **e** The survival analysis of HCC patients (age ≥ 65) by OS-related lncRNA promoter methylation model. **f** The survival analysis of HCC patients (age < 65) by OS-related lncRNA promoter methylation model. **g** The survival analysis of HCC patients (female) by OS-related lncRNA promoter methylation model. **h** The survival analysis of HCC patients (male) by OS-related lncRNA promoter methylation model. **i** The survival analysis of HCC patients with high T stage by OS-related lncRNA promoter methylation model. **j** The survival analysis of HCC patients with low T stage by OS-related lncRNA promoter methylation model. **k** The survival analysis of HCC patients with tumor-free status by OS-related lncRNA promoter methylation model

In addition, further analysis suggested that this OS-related model could also effectively divide patients into high-risk and low-risk groups for the following categories (Fig. 3): clinical stage (Fig. 3a, b), pathological grade (Fig. 3c, d), age (Fig. 3e, f), gender (Fig. 3g, h), tumor T stage (Fig. 3i, j), tumor-free survival (Fig. 3k). The survival rate of each high-risk group was significantly lower than that of the low-risk group. The clinical stage and tumor T stage shown in Fig. 3 is according to the AJCC eighth edition; the pathological grade is in the light of the Edmondson classification.

**Fig. 4:**
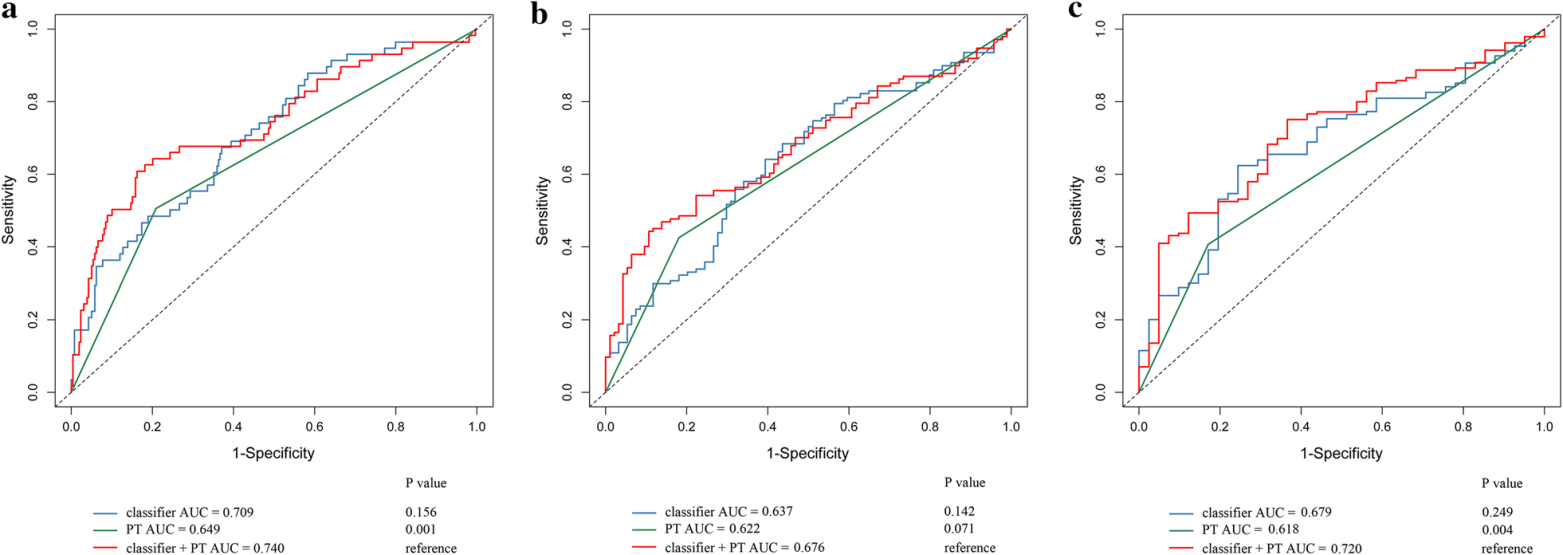
ROC curve analysis of the OS-related lncRNA promoter methylation model combined with T category of AJCC stage. **a** OS-related lncRNA promoter methylation model combined with T stage to evaluate 1-year OS of HCC patients. **b** OS-related lncRNA promoter methylation model combined with T stage to evaluate 3-year OS of HCC patients. **c** OS-related lncRNA promoter methylation model combined with T stage to evaluate 5-year OS of HCC patients

Additionally, COX univariable and multivariable regression analyses were shown in Table 3, which suggested that the tumor T category of AJCC stage (HR 2.166, 95% CI 1.490–3.148, P < 0.001) and OS-related lncRNA model (HR 2.014, 95% CI 1.378–2.942, P < 0.001) were independent risk factors that affected the OS of HCC patients. Thus, the OS-related lncRNA model combined with T category of AJCC stage to evaluate the overall survival in 1-, 3-, and 5-year (Fig. 4) with its AUC values were higher than that of the OS-related model and T category alone.

**Table 3:** COX regression analysis of lncRNA survival model and relationship between clinicopathological features and OS of HCC

Variables	Univariable analysis		Multivariable analysis	
	HR (95% CI)	P value	HR (95% CI)	P value
Gender	1.205 (0.846–1.714)	0.304		
Age	1.012 (0.998–1.025)	0.097		
Tumor stage (G3/4 vs G1/2)	1.044 (0.729–1.496)	0.815		
T category (T3/4 vs T1/2)	2.483 (1.756–3.517)	< 0.001	2.166 (1.490–3.148)	< 0.001
Pathological grade (III + IV vs I + II)	2.382 (1.649–3.443)	< 0.001		
Vascular infiltration (with vs without)	1.358 (0.900–2.051)	0.148		
lncRNA survival model (high-risk vs low-risk)	2.131 (1.500–3.028)	< 0.001	2.014 (1.378–2.942)	< 0.001

### Development and evaluation of the vascular invasion-related lncRNA promoter methylation model

**Fig. 5:**
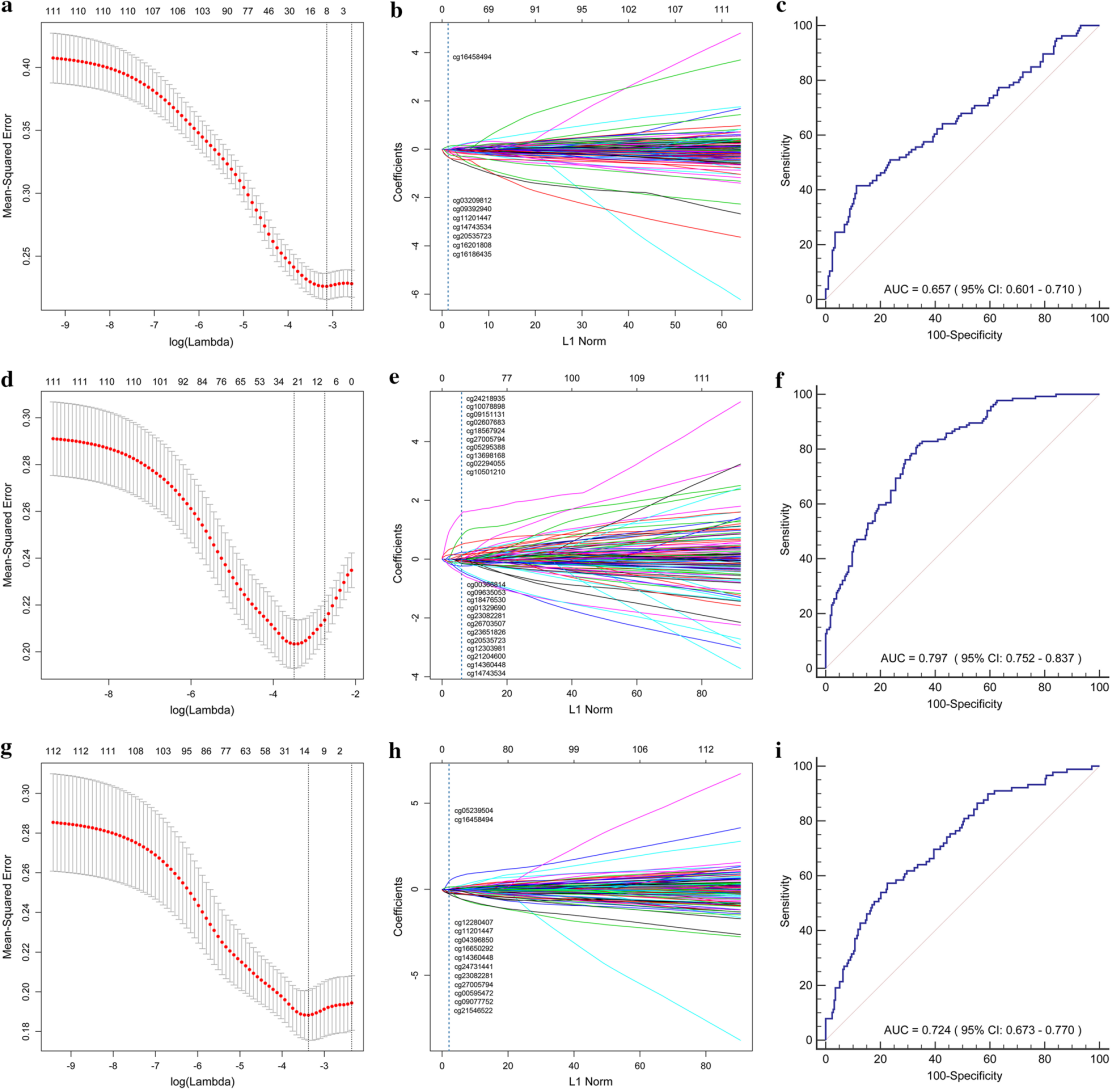
Development and evaluation of the vascular infiltration, pathological grade, and clinical stage-related lncRNA promoter methylation model. **a** Vascular infiltration-related lncRNA promoter methylation sites were screened by LASSO-COX method. **b** The coefficients of each methylation sites were determined by LASSO-COX method. **c** ROC curve analysis of vascular infiltration-related lncRNA promoter methylation model to evaluate vascular infiltration in HCC patients. **d** Pathological grade-related lncRNA promoter methylation sites were screened by LASSO-COX method. **e** The coefficients of each methylation sites were determined by LASSO-COX method. **f** ROC curve analysis of pathological grade-related lncRNA promoter methylation model to evaluate pathological grade in HCC patients. **g** Clinical stage-related lncRNA promoter methylation sites were screened by LASSO-COX method. **h** The coefficients of each methylation sites were determined by LASSO-COX method. **i** ROC curve analysis of clinical stage-related lncRNA promoter methylation model to evaluate clinical stage in HCC patients

In this section, we utilized LASSO-Logistic to select variables and determine the coefficients, and finally derived the vascular invasion-related lncRNA promoter methylation model (Fig. 5a, b).

The vascular invasion-related model was constructed as follows:

$$ \begin{aligned} & - \, 1. 1 6 9\, - \,0.0 1 6\, \times \,{\text{E}}\_{\text{cg}}0 3 20 9 8 1 2\, - \,0.0 2 9\, \times \,{\text{E}}\_{\text{cg}}0 9 3 9 2 9 40 \\ & \quad - \,0.0 5 6\, \times \,{\text{E}}\_{\text{cg112}}0 1 4 4 7\, - \,0. 10 1\, \times \,{\text{E}}\_{\text{cg14743534}}\, \\ & \quad - \,0. 2 80\, \times \,{\text{E}}\_{\text{cg16186435}}\, - \,0. 2 7 2\, \times \,{\text{E}}\_{\text{cg162}}0 1 80 8\\ & \quad + \,0.0 9 5\, \times \,{\text{E}}\_{\text{cg16458494}}\, - \,0. 1 3 6\, \times \,{\text{E}}\_{\text{cg2}}0 5 3 5 7 2 3. \\ \end{aligned} $$

The AUC value of the vascular invasion -related lncRNA promoter methylation model was 0.657 (95% CI 0.601–0.710, sensitivity: 41.51%, specificity: 88.73%, accuracy: 72.58%) and is plotted in Fig. 5c, which suggested that this model possessed a certain degree of accuracy.

### Development and evaluation of the pathological grade-related lncRNA promoter methylation model

Similarly, there were 22 methylation sites associated with pathological grade (Fig. 5d, e).

The pathological grade-related model was constructed as follows:

$$ \begin{aligned} & - \,0. 1 10\, - \,0.0 1 5\, \times \,{\text{E}}\_{\text{cg}}00 3 6 6 8 1 4\, - \,0.0 5 4\, \times \,{\text{E}}\_{\text{cg}}0 1 3 2 9 6 90\, + \,0.00 8\, \times \,{\text{E}}\_{\text{cg}}0 2 2 9 40 5 5\\ & \quad + \,0. 1 9 9\, \times \,{\text{E}}\_{\text{cg}}0 2 60 7 6 8 3\, + \,0.0 4 6\, \times \,{\text{E}}\_{\text{cg}}0 5 2 9 5 3 8 8\, + \,0. 4 5 3\, \times \,{\text{E}}\_{\text{cg}}0 9 1 5 1 1 3 1\\ & \quad - \,0.0 3 8\, \times \,{\text{E}}\_{\text{cg}}0 9 6 3 50 5 3\, + \,0. 6 2 6\, \times \,{\text{E}}\_{\text{cg1}}00 7 8 8 9 8\, + \,0.00 7\, \times \,{\text{E}}\_{\text{cg1}}0 50 1 2 10 \\ & \quad - \,0. 2 2 2\, \times \,{\text{E}}\_{\text{cg123}}0 3 9 8 1\, + \,0.0 4 2\, \times \,{\text{E}}\_{\text{cg13698168}}\, - \,0. 3 3 8\, \times \,{\text{E}}\_{\text{cg1436}}0 4 4 8\\ & \quad - \,0. 4 1 8\, \times \,{\text{E}}\_{\text{cg14743534}}\, - \,0.0 5 1\, \times \,{\text{E}}\_{\text{cg1847653}}0\, + \,0. 1 4 9\, \times \,{\text{E}}\_{\text{cg18567924}} \\ & \quad - \,0. 1 3 3\, \times \,{\text{E}}\_{\text{cg2}}0 5 3 5 7 2 3\, - \,0. 2 4 9\, \times \,{\text{E}}\_{\text{cg212}}0 4 600\, - \,0.0 70\, \times \,{\text{E}}\_{\text{cg23}}0 8 2 2 8 1\\ & \quad - \,0. 1 2 7\, \times \,{\text{E}}\_{\text{cg23651826}}\, + \, 1. 3 9 9\, \times \,{\text{E}}\_{\text{cg24218935}}\, - \,0.0 8 2\, \times \,{\text{E}}\_{\text{cg267}}0 3 50 7\\ & \quad + \,0.0 9 6\, \times \,{\text{E}}\_{\text{cg27}}00 5 7 9 4. \\ \end{aligned} $$

The AUC value of the pathological grade-related lncRNA promoter methylation model was 0.797 (95% CI 0.752–0.837, sensitivity: 81.34%, specificity: 66.52%, accuracy: 72.02%) and was plotted in Fig. 5f, which suggested that this model possessed significant accuracy.

### Development and evaluation of the clinical stage-related lncRNA promoter methylation model

There were 13 methylation sites associated with clinical stage (Fig. 5g, h).

The clinical stage-related model was constructed as follows:

$$ \begin{aligned} & - 1. 9 4 3\, - \,0. 20 9\, \times \,{\text{E}}\_{\text{cg}}00 5 9 5 4 7 2\, - \,0.0 1 5\, \times \,{\text{E}}\_{\text{cg}}0 4 3 9 6 8 50\, + \,0. 3 4 8\, \times \,{\text{E}}\_{\text{cg}}0 5 2 3 9 50 4\\ & \quad - \,0. 2 1 7\, \times \,{\text{E}}\_{\text{cg}}0 90 7 7 7 5 2\, - \,0.0 1 5\, \times \,{\text{E}}\_{\text{cg112}}0 1 4 4 7\, - \,0.00 7\, \times \,{\text{E}}\_{\text{cg1228}}0 40 7\\ & \quad - \,0.0 4 2\, \times \,{\text{E}}\_{\text{cg1436}}0 4 4 8\, + \,0. 1 1 8\, \times \,{\text{E}}\_{\text{cg1645849}}\, - \,0.0 30\, \times \,{\text{E}}\_{\text{cg1665}}0 2 9 2\\ & \quad - \,0. 2 4 1\, \times \,{\text{E}}\_{\text{cg21546522}}\, - \,0. 1 5 3\, \times \,{\text{E}}\_{\text{cg23}}0 8 2 2 8 1\, - \,0. 1 3 9\, \times \,{\text{E}}\_{\text{cg24731441}} \\ & \quad - \,0. 1 7 2\, \times \,{\text{E}}\_{\text{cg27}}00 5 7 9 4. \\ \end{aligned} $$

The AUC value of the clinical stage-related lncRNA promoter methylation model was 0.724 (95% CI 0.673–0.770, sensitivity: 57.30%, specificity: 77.47%, accuracy: 72.22%) and was plotted in Fig. 5i, which suggested that this model possessed significant accuracy.

### Functional analysis of the screened vascular invasion-related lncRNA related to promoter methylation sites

**Fig. 6:**
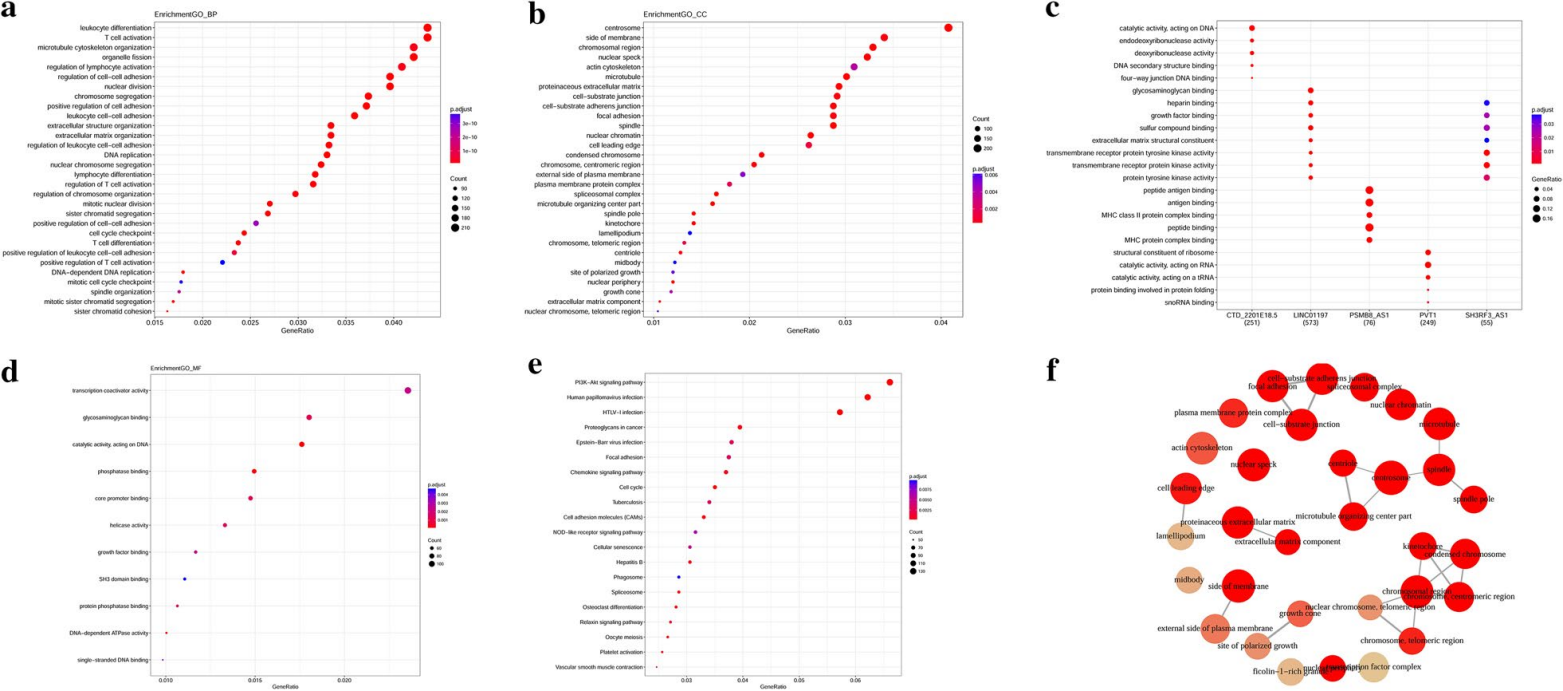
The functional analysis of the lncRNAs corresponding to vascular infiltration-related lncRNAs promoter methylation model. **a** GO-BP analysis of lncRNA target gene. **b** GO-CC analysis of lncRNA target gene. **c** GO analysis after grouping target genes of vascular infiltration-related lncRNA. **d** GO-MF analysis of lncRNA target gene. **e** KEGG-pathway analysis of lncRNA target gene. **f** Molecular functional network of lncRNA target genes

Functional analysis of vascular invasion-related lncRNA related to promoter methylation sites was performed via GO/KEGG pathway enrichment analysis. The mainly GO enrichment term was the biological process (BP) (Fig. 6a) which suggested that the vascular invasion-related lncRNA involving leukocyte differentiation, T cell activation, microtubule cytoskeleton organization, organelle fission, regulation of lymphocyte activation, regulation of cell–cell adhesion, and nuclear division. In Fig. 6b suggested that the centrosome, side of the membrane, chromosomal region were enriched GO terms related to the cellular components (CC). In Fig. 6d, transcription coactivator activity composed the cellular molecular function (MF). The results of the KEGG pathway were plotted in Fig. 6e, which suggested that lncRNA corresponding to vascular invasion-related methylation sites affected HCC via the PI3K/Akt signaling pathway.

### Verification of the vascular invasion-related lncRNA promoter methylation model

**Fig. 7:**
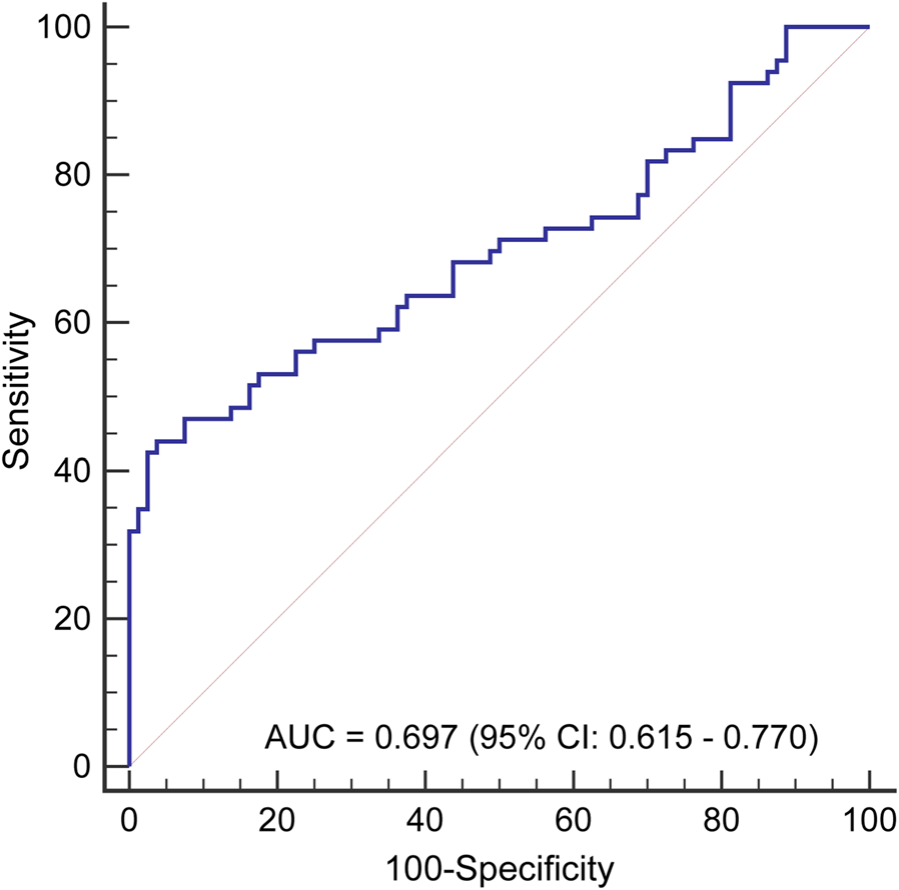
Verification of the accuracy of vascular infiltration-related lncRNA promoter methylation model by using HCC tissues

In MS-PCR, a total of 146 HCC tissues were utilized to detect the degree of methylation of each site and to verify the accuracy of the vascular invasion-related lncRNA promoter methylation model. The AUC value (Fig. 7) of the vascular invasion-related model was 0.697 (95% CI 0.615–0.770, sensitivity: 43.94%, specificity: 96.25%, accuracy: 72.60%), which is consistent with our previous bioinformatics assay.

## Discussion

Previously, the role of protein-coding genes in the pathogenesis of HCC has been the focus of research in the field of oncology. However, with the advancement of high-resolution microarrays and massively parallel sequencing technologies, ncRNAs that do not encode proteins have been proven to possess many kinds of biological functions [21, 22]. The current study utilized biomedical statistical research methods based on bioinformatics. On the basis of statistical significance, inferring possible medical mechanisms and molecular regulatory mechanisms provides the whole perspective for the study of a biomedical phenomenon to design more specific molecular biological experiments.

Although the function of HCC-related lncRNA expression regulation and molecular mechanisms that are caused by its promoter methylation remains unclear, some previous studies have yielded similar results. Tang et al. [23] found that lncRNA CRNDE promotes HCC cells proliferation by affecting the PI3K/Akt/GSK3β-Wnt/β-catenin signaling pathway, and CRNDE is closely correlated with a poor prognosis of HCC patients. Hou et al. [24] utilized 5 lncRNAs (CTD-2116N20.1, AC012074.2, RP11-538D16.2, LINC00501, and RP11-136I14.5) to construct a prognostic model of HCC patients, with a C-index of 0.701. Another lncRNA SRHC has been proven to have the ability to inhibit HCC cells proliferation with low transcription levels in HCC tissues due to promoter methylation [25]. Braconi et al. [26] found that miR-29a promotes excessive transcription in HCC tissues by inhibiting the methylation of the lncRNA MEG3 promoter methylation to stimulates proliferation of HCC cells. In addition, another study completed by utilizing bioinformatics methods found that 6 lncRNAs (CECR7, LINC00346, MAPKAPK5-AS1, LOC338651, FLJ90757, LOC283663) were significantly correlated with the OS of HCC patients [27]. LINC00346 is one of them, which happens to be the lncRNA corresponding to the methylation site cg13698168 we identified. Although this is a coincidence, it also reflects the accuracy of the methylation sites we screened for previously.

The angiogenesis and vascular invasion of HCC have always been research hotspots, and anti-angiogenesis has been applied in clinical practice and has achieved certain curative effects. However, the efficacy of these drugs still needs to be improved. Thus, it is necessary to find new targets of diagnosis or to improve the efficacy of existing drugs [28]. Recent studies suggested that lncRNA FEZF1-AS1 [29], the signaling axis of lncRNA n335586/miR-924/CKMT1A [30], lncAKHE [31], and the signaling axis composed of PVT1/EZH2/miR-214 [32] were involved in the regulation of invasion of HCC via many kinds of signaling pathway. The methylation of a lncRNA promoter directly affects its transcription level, which inevitably has a decisive influence on the downstream lncRNA function. The methylation site of the promoter associated with HCC vascular invasion in this study lacks in-depth mechanistic research. The results of this study can provide ideas for further research. Moreover, most of the screened CG sites and their corresponding miRNAs have not been reported in in-depth studies related to vascular invasion, which reflects the innovation of this research and provides the whole perspective for future research.

The commonality of these studies is that one of the prerequisites for the HCC-related lncRNAs to contribute to their abnormal behavior is through promoter methylation levels. Therefore, finding HCC-related lncRNA promoter methylation sites is essential to explore the upstream mechanism of lncRNA regulation of HCC, which can lead to understanding the occurrence and development of HCC and exploring new therapeutic targets. All of these efforts are new and significant in terms of finding new tumor-related markers.

## Conclusion

In summary, the biological phenomenon of lncRNA promoter methylation is closely correlated with the occurrence and development of HCC in many aspects, including overall survival, vascular invasion, pathological grade, and clinical stage. We utilized bioinformatics methods to screen for these lncRNA promoter methylation sites that have become new potential targets for the diagnosis, therapy options, and the prognosis of HCC patients.

## Data Availability

Data of clinical characteristic, expression profile of lncRNA, DNA methylation, and mRNA expression profile were taken from the Cancer Genome Atlas (TCGA). Data with the following two conditions were excluded: not diagnosed with HCC and loss of relevant data. A total of 367 HCC samples and 50 cancer adjacent samples were included in this study for subsequent analysis. We collected 146 cancer tissues from HCC patients who underwent surgical treatment at our hospital between June 2017 and April 2018.

## Abbreviations

RNA: ribonucleic acid
DNA: deoxyribonucleic acid
ncRNA: noncoding RNA
lncRNA: long noncoding RNA
mRNA: messenger RNA
HCC: hepatocellular carcinoma
PLC: primary liver cancer
TCGA: the Cancer Genome Atlas
OS: overall survival
MS-PCR: methylation-specific polymerase chain reaction
ROC: receiver operating characteristic
AUC: area under the ROC curve
td-AUC: time-dependent AUC
LASSO: the least absolute shrinkage and selection operator
